# Supplementary material for: The Vaginally Exposed Extracellular Vesicle of Gardnerella vaginalis Induces RANK/RANKL-Involved Systemic Inflammation in Mice
Source: Microorganisms. 2025 Apr 21;13(4):955. doi: 10.3390/microorganisms13040955 (PMC12029968; doi:10.3390/microorganisms13040955)
Supplement: Supplementary file 1 [file microorganisms-13-00955-s001.zip › microorganisms-3552643-supplementary.pdf]

[Supporting information]

The vaginally exposed extracellular vesicle of *Gardnerella vaginalis* induces RANK/RANKL-involved systemic inflammation in mice

Table S1. Primer used for qPCR

		Primer sequence
G. vaginalis	Forward	ACCTGGGCTTGACATGTGCCT
	Reverse	CATGCACCACCTGTGAACCTG
16S rRNA gene	Forward	TCGTCGGCAGCGTCAGATGTGTATAAGAGACAGGTGCCAGCMGCCGCGGTAA
	Reverse	GTCTCGTGGGCTCGGAGATGTGTATAAGAGACAGGGACTACHVGGGTWTCTAAT
Osteocalcin	Forward	TGAGGACCATCTTCTGCTCA
	Reverse	TGGACATGAAGGCTTTGTCA
Osteoprotegerin	Forward	AGCCATTGCACACCTCAC
	Reverse	CGTGGTACCAAGAGGACAGAGT
RANK	Forward	ATCTCGGACGGTGTTGCAG
	Reverse	TCTTTCATTCCAGGTGTCCAAGTA
RANKL	Forward	AACCAAGATGGCTTCTATTACC
	Reverse	AAGGGTTGGACACCTGAATG
TRAP	Forward	GCTACTTGCGGTTTCACTATGGA
	Reverse	TGGTCATTTCTTTGGGGCTTATCT
TNF-α	Forward	AGCCCACGTAGCAAACCACCAA
	Reverse	ACACCCATTCCCTTCACAGAGCAAT
IL-6	Forward	CAACGATGATGCACTTGCAGA
	Reverse	GTGACTCCAGCTTATCTCTTGGT
GAPDH	Forward	TGCAGTGGCAAAGTGGAGAT
	Reverse	TTTGCCGTGAGTGGAGTCATA

Table S2. LC-MS-MS data of EV A and B proteins

	NCBI BLAST	Protein	Monoisotopic mass (M_r)	Matched peptide number	Protein sequence coverage (%)
A	WP_013399750.1	type I polyketide synthase	343340	41	27
B	WP_142369470.1	hypothetical protein, partial of *Klebsiella oxytoca*	73897	18	2

[Methods]

Behavioral tasks

Open Field Test (OFT) - The OFT was used to evaluated the effects on anxiety-related behaviors. In the test, mice were placed in an open arena divided into a central area and a peripheral area of a chamber (40 cm × 40 cm). The animal’s movement and behavior were recorded for 10 min. During the test, the distance traveled, the time spent in the center, and the velocity were measured using the EthoVision XT software.

Elevated plus maze task (EPMT) - The EPMT was used to evaluate anxiety-related behaviors. It consists of a plus-shaped platform with two open arms and two closed arms (30 × 6 × 20 cm walls) that were elevated at a height of 40 cm above the bottom. Mice were placed on the central platform facing one of the open arms. The animal’s behavior was recorded for 5 min. The percentage of time spent in open arms and the number of entries into open arms were measured and corrected by total time or entries in open plus closed arms, respectively.

Tail Suspension Test (TST) - The TST was used to evaluate depression-related behaviors. During the test, mice were suspended by its tail using a clip with their body positioned vertically. The amount of immobile time was recorded for 6 min. The percentage of total immobility time was quantified.

Y-maze (YMT) - The YMT was performed in a horizontal maze made of black-coloured acryl and positioned at equal angles (length, 40 cm; width, 3 cm; and wall height, 12 cm). Mice were placed at the end of the arm and allowed to move freely through the maze during 8-min sessions. Arm entry sessions were recorded when the hind paws of the rat were completely placed in the arm. A spontaneous alternation was defined as entries into all three arms on consecutive choices. The ratio (%) of actual to possible alternations was calculated.

Novel object recognition test (NORT) - The NOR task was performed in in a square box (45 × 45 × 50 cm). For the first trial, a mouse was placed in the box containing two identical objects and the frequency of touching each object was recorded for 10 min. In the second trial conducted 24 h after the first trial, a mouse was placed in the box containing one of the old objects used in the first trial and a new object. Novel object recognition was calculated as the ratio of the number of times touching the new object to the sum of the touching frequencies.
